# Properties of Injection Molded Biocomposites Reinforced with Wood Particles of Short-Rotation Aspen and Willow

**DOI:** 10.3390/polym12020257

**Published:** 2020-01-22

**Authors:** Anuj Kumar, Tuula Jyske, Veikko Möttönen

**Affiliations:** 1 Natural Resources Institute Finland (Luke), Production Systems, Tietotie 2, FI-02150 Espoo, Finland; tuula.jyske@luke.fi; 2 Natural Resources Institute Finland (Luke), Production Systems, Yliopistokatu 6, FI-80100 Joensuu, Finland; veikko.mottonen@luke.fi

**Keywords:** short-rotation, aspen, willow, injection molding, biocomposite, tensile strength, bending strength, microstructure behavior

## Abstract

Injection molded biocomposite specimens were prepared by using four different weight percentages, i.e., 10%, 20%, 30%, and 40% of aspen (*Populus tremula* L.) and willow (*Salix caprea* L.) wood particles in a biopolymeric matrix. Dog-bone test specimens were used for testing the physical, mechanical, and thermal properties, and microstructure of biocomposites. The tensile and bending strength changed with the change in weight percentages of wood particles and the bending stiffness increased with the increasing weight percentage of wood. In Brinell hardness, similar changes as a function of wood particle weight percentage were shown, and a relationship between hardness and tensile strength with wood content was also investigated. The prepared biocomposites could be an alternative for plastic-based materials and encourage the use of fast growing (aspen and willow) wood from short-rotation forests in biocomposites.

## 1. Introduction

Future social and economic development globally depends on our success in mitigating climate change by transforming our dependence on finite fossils fuels into use of sustainable resources. The key concept of the circular economy is to reduce waste levels and increase the utilization of side-streams and low-value wood that can be transformed into biocomposites and other value added products from the view point of wood products cluster [1,2,3]. Biomass materials, such as wood, represent environmentally friendly alternatives for fossil resources that play a key role while also turning societies towards sustainable and circular bioeconomy [4,5]. Various types of wood products, such as engineered wood, and wood-based panels incorporate wood, as raw material in varied forms, into industrial applications that are manufactured by using effective processing methods [6,7]. Such manufacturing methods are able to utilize wood with inconvenient shapes, such as branches and side-streams, or fast-growing, small-diameters species (i.e., aspen or willow), being otherwise difficult to convert into valuable products. Currently, the fast-growing coppice species aspen and willow are either used for energy generation or particleboard production [8]. Fast-growing species could also be used for producing higher added value design biocomposites. Biocomposites are defined as materials where the polymeric matrix or resin and the reinforcement (fibers, particles, powder, etc.) are entirely made from renewable resources. In recent years, they have attracted considerable interest due to their sustainability with great potential to become eco-friendly, biodegradable substitutes for petroleum-based polymeric matrices [4,9].

Many types of natural origin fibers are predominately consumed in biocomposites production. Flax, hemp, jute, coir, cotton, sisal, kenaf, silk, and bamboo fibers are the most explored cellulosic fibers [10,11,12]. Migneault et al. [13] studied the effects of wood fiber origin, proportion, and chemical composition on the properties of wood-plastic composites (WPC). Interestingly, pulp and paper sludge-based WPC showed better overall properties when compared with the other raw materials. Csikós et al. [14] fabricated the poly(lactic) acid (PLA) and Filtracel EFC 1000 (Rettenmaier and Söhne GmbH) wood fiber composite and studied the surface of wood fibers on the interfacial bonding between wood fibers and polymer matrix. Porebska et al. [15] found that the polymer matrix influenced the properties of wood polymer composites when they prepared cellulose fiber reinforced composites with polypropylene, polystyrene, polyoxymethylene, acrylonitrile butadiene styrene, polyester resin, and PLA with different contents of cellulose fibers, by using injection molding process. The size of wood fibers could influence the processing and properties of wood polymer composites [16]. The saw dust of spruce wood could be potential filler for high-density polyethylene (HDPE)-based composite [17]. The properties of wood fibers are dependent on the species, contents, defects, physical, and mechanical properties, as well as the interaction of a fiber with the polymer in the wood polymer composites [18]. Hardwood and softwood fibers could both be suitable for wood polymer composites while using injection molding technique with different particle size and dimension [19]. Surface treatment of wood fiber with alkali improved the compatibility with polymer matrices by creating rough surface, cavities, and much interspace between smaller fibrils [20]. Effah et al. [21] fabricated wood polymer composites while using different wood species, such as pine, eucalyptus, black wattle, long-leaved wattle, port jackson and beefwood, with a low density polyethylene (LDPE) matrix. The different wood fibers interacted differently with polymer matrix due to the differences in chemical and physical properties. Recently, fast-growing willow (*Salix viminialis*) and high-density polyethylene (PEHD)-based injection molded composites were compared with the properties other commercial Lignocel C-120 fibers-based composite [22]. 

Short-rotation forest (SRF) plantations are gaining attention in many countries, especially when grown for energy production [23]. Among the different fast-growing hardwoods that were proposed for energy uses, willow (*Salix*) is one of the few that has been planted commercially to a significant extent in the European Union (EU). In Northern Europe, it presents the advantages of high productivity in Nordic conditions [23,24]. European aspen (*Populus tremula* L.) and hybrid aspen (*Populus tremula* L. x *P. tremuloides* Michx.) have proved to be one of the fastest growing deciduous tree species in Nordic countries, with successful breeding and cultivation of hybrid aspen since the early twentieth century [25]. In Finland, aspen is mainly used for paper and energy production [26,27].

The aim of this study was to investigate the effect of using the short-rotation wood particles as filler on the physical and mechanical properties and microstructure of injection molded biocomposite. The physical (density, color change, and water uptake), chemical (Fourier transform infrared spectrophotometer (FTIR)), and mechanical properties (tensile strength, bending strength, and stiffness), as well as microstructure of biocomposites were evaluated. Further, the relationship between Brinell hardness and tensile strength of biocomposites as a function of wood particles content was also investigated.

## 2. Materials and Methods

### 2.1. Preparations of Wood Particles

Short-rotation European aspen (*Populus tremula* L.) and willow (*Salix caprea* L.) trees (two aspen and 19 willow stems) were harvested in Tuusula (60°33′09″N, 24°58′06″E; 43 m a.s.l.) in southern Finland. The tree height and stem diameter at butt (0 m), breast height (1.3 m), and 6 m (aspen only) were measured for each tree (Table 1).

**Figure 1:**
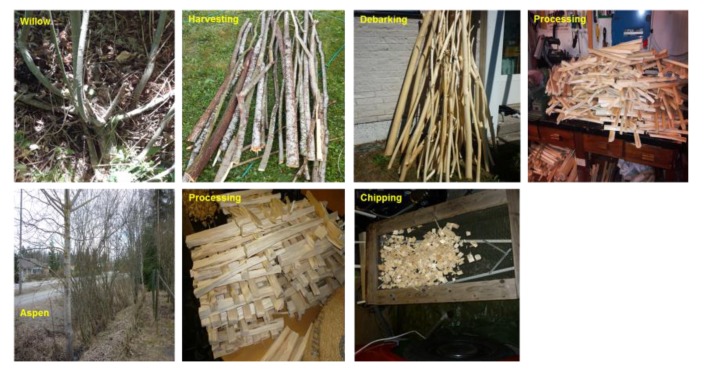
Harvesting and chipping process of short-rotation aspen and willow.

The stems were then sawn into smaller blocks, from which bark, branches, larger knots, and defects and whorls were removed. The blocks were converted into wood chips while using a lab-based chipping machine, as described in Figure 1. The chips were dried with warm air at a room temperature for two to three weeks to stabilize the moisture content. The corn starch-based polylactic acid (PLA) was purchased from Sigma Aldrich (Helsinki, Finland) as a biopolymer matrix for biocomposites fabrication. 

Aspen and willow chips were milled with Fritsch Pulverisette mill (Helsinki, Finland). A two-step milling process was applied, where the second, fast rotating cutting blade followed the first slowly rotating crushing blade. Against the second blade, a sieve with 2.0 mm openings was used. The particle size varied from 0.2 mm × 1.0 mm × 2.0 mm to 0.5 mm × 2.0 mm × 6.0 mm. The milled powder was dried for at least 4 h at 105 °C, and the mixtures with natural binder were made at 80 °C to avoid moisture absorption to the raw materials. The wood particles of both species were mixed to natural binder in 10%, 20%, 30%, and 40% based on dry weight percentage of PLA before molding process. 

### 2.2. Injection Molding Process

**Figure 2:**
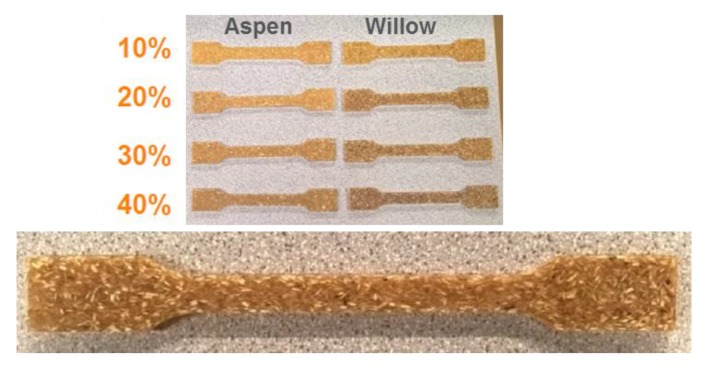
Dog-bone shaped biocomposite test samples of aspen and willow.

The test specimens (Figure 2) of aspen and willow wood with PLA matrix were molded at industrial scale with a twin-extruder (Engel ES 200/50 HL, Eschweiler, Germany), according to ASTM D638 standards. Twenty tensile bars of each weight percentages of wood in biocomposites were prepared for tests of tensile, bending strength, and Brinell hardness. All of the specimens were conditioned at 20 °C and 65% relative humidity for 48 h prior to testing. The nominal dimensions of specimens were, as follows: gauge length 80 mm, width 10 mm, and thickness 4 mm. The following temperature settings were used for injection molding process: Feed zone: 185 °C, compression zone: 190 °C, homogenizing zone: 190 °C, machine nozzle: 195 °C, and mold temperature: 30 °C. The gravimetric method measured the density of all the prepared biocomposites.

### 2.3. Mechanical Properties

The mechanical properties were tested while using Zwick Z050 (Kennesaw, GA, USA) material testing machine. Tensile strength was measured according to EN ISO 527, bending strength (*σ_w_*, MPa), and modulus of elasticity (*E_w_*, GPa) were measured according to EN ISO 178, and Brinell hardness (HB, MPa) was measured according to EN 1534 [28].

### 2.4. Color Measurement

The surface reflectance spectrum was measured from the range of 8 mm in diameter of each specimen in the visible light wavelength range 360–740 nm while using a Konica Minolta CM-2600d spectrophotometer (New Jersey, NY, USA) Spectral data was converted to CIEL*a*b* color coordinates using 2° standard observer and D65 light source for lightness (L*), redness (a*), and yellowness (b*), according to CIEL*a*b* color space (ISO 11664-4:2008). For each sample group, the mean and standard deviations of the color coordinates were calculated. 

### 2.5. Microstructures of Biocomposites

Small specimens (ca. 20 mm × 5 mm × 4 mm) having a trapezoid-shaped head were cut from the biocomposite samples by using a saw and a razor blade. Semi-thin sections (ca. 5–6 µm thick) were cut from trapezoids by using glass knives in a rotary microtome (Leica RM2265, Leica Microsystems, Wetzlar, Germany). The sections were stained with an aqueous solution of 0.1% toluidine blue, air-dried, and then mounted in Ultrakitt M540 mountant (TAAB, Reading, UK). Optical microscope images of sections were taken by using a digital camera (MicroPublisher 3.3 RTV, QImaging, Surrey, BC, Canada; 6.6 PL-B686CF-KIT, PixeLINK, Ottawa, ON, Canada) that was attached to a light microscope (Olympus BX60 or Olympus BX50) at 10X -magnification and with a resolution of 0.343 µm/pixel.

### 2.6. FTIR Measurement

Fourier transform infrared spectrophotometer (Shimadzu Cooperation, Kyoto, Japan IRPrestige-21/IRAffinity-1/FTIR-8000 series) coupled with IRsolution software to control them and data processing used in this work. Semi-thin layers were cut from biocomposites, a razor blade, and then dried at 60 °C for two hours. The prepared samples were scanned while using Attenuated Total Reflection (ATR) setup in the absorbance range of 400–4000 cm^−1^ with a scanning rate of 2 cm^−1^ and 50 scans per run.

### 2.7. Water Absorption 

Three specimens of each biocomposite samples with nominal dimensions of 32 mm × 20 mm × 4 mm were immersed into water for 24 h and for four days and weight change was measured to calculate the water absorption percentage.

### 2.8. Statistical Analysis

One-way analysis of variance (ANOVA) at P < 0.05 level was performed to identify the statistical difference between the control PLA sample and wood particles reinforced biocomposite samples by using Microcal Origin statistical software.

## 3. Results and Discussion

### 3.1. Density and Water Absorption

The density of pure PLA injection molded composite varied between 1.22 to 1.26 g/cm^3^. The density of biocomposite increased with an increasing weight percentage of wood particles (Table 2). The density of aspen-based biocomposite was increased from 1.22 g/cm^3^ (wood content 10%) to 1.32 g/cm^3^ (wood content 40%), while the pure biopolymer possesses density between. The willow-based biocomposite showed higher density in comparison to aspen-based biocomposite.

The stability of biocomposite in humid condition or water contact is a very essential characteristic that is required for several applications. Table 2 shows the water absorption of biocomposites after being immersed into water for 24 h and four days. The water absorption capacity of biocomposite increased with increase in weight percentage of wood particles. After 24 h, the 10% aspen sample showed 0.33% water absorption, while the 40% aspen sample reached 0.90% water absorption. Similar trend was observed for willow-based biocomposites, but with higher average water absorption than that of aspen-based biocomposites. Increasing trend of water uptake with wood particle content was also observed after four days of immersion. However, the water absorption was significantly higher, i.e., 2.79% and 6.20% for 40% aspen and willow samples, respectively. These results are in accordance to studies that were reported in literature, where wood-based material was used in biocomposite preparation [29,30].

### 3.2. Tensile Testing

**Figure 3:**
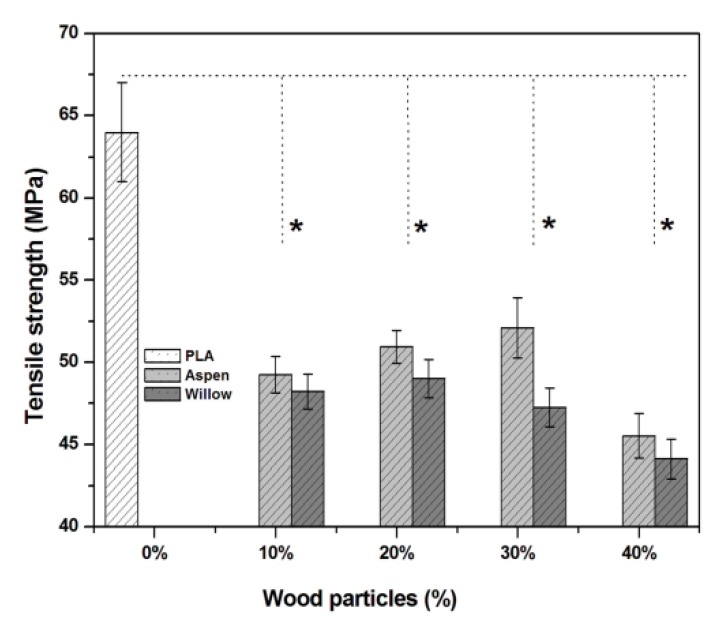
Tensile strength comparison between different species. (* Significant difference at *p* < 0.05)

To evaluate the effect of reinforcement by wood particles on the PLA matrix, the mechanical properties of biocomposites were determined. The tensile strength of PLA was 64 ± 3 MPa. The addition of 10% of wood particles significantly reduced the tensile strength by 26% to 49 ± 2 MPa, as shown in Figure 3. This might be due to the random distribution of wood particles in biopolymer matrix, which has created different load transfer points within biocomposite due to packing frication. Tensile strength increased with further increase in wood particle content up to 30. However, increasing the wood particles content beyond 30% was found to have a negative impact on bond formation between polymer matrix and wood particles, thus creating weak interfacial regions [31]. Earlier studies also showed similar behavior in the reduction of tensile strength of wood particles (flour and fibers) reinforced PLA-based biocomposites with increasing wood content [32,33,34]. 

### 3.3. Bending Strength and Stiffness

**Figure 4:**
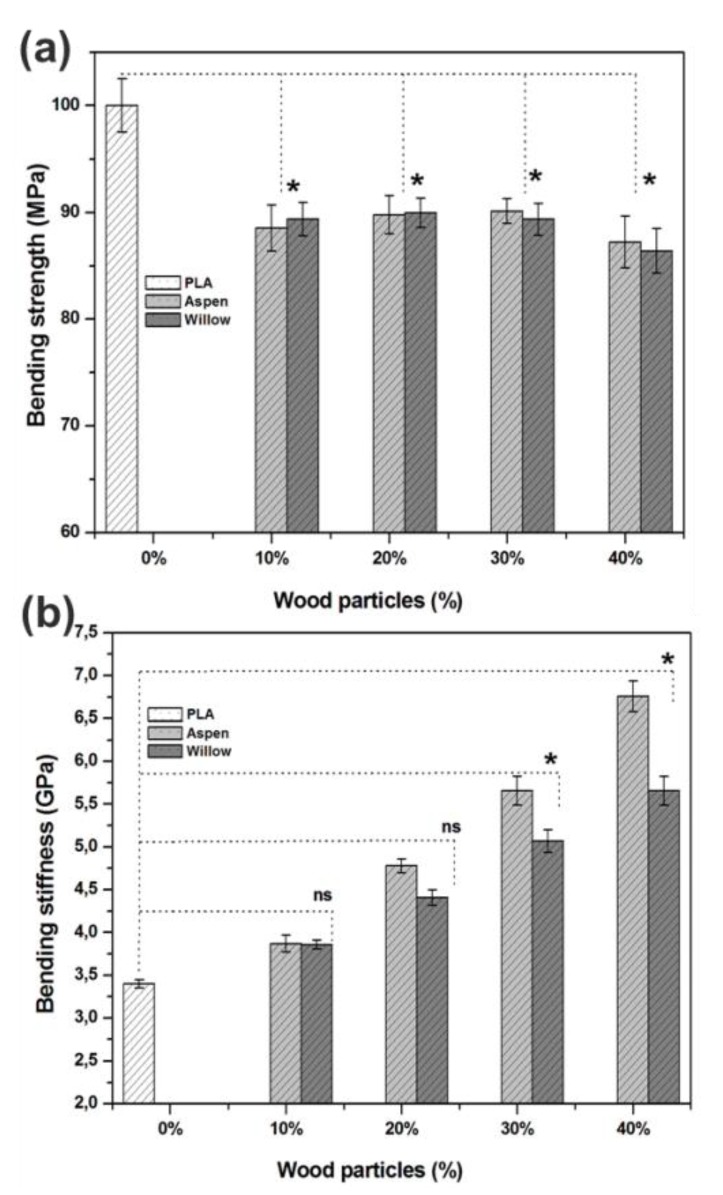
Comparison of the bending properties between the PLA and biocomposite samples: (**a**) Bending strength and (**b**) Bending stiffness. (* significant difference at *p* < 0.05 and ns not significant).

The pure biopolymer of this study showed a strong bending strength of 100 MPa, which is comparable to the bending strengths that were reported for other biopolymers, such as PLA and polyester resin [15,21]. Figure 4a shows the bending strength of the prepared biocomposites. The bending strengths of wood particle-reinforced biocomposites were significantly decreased as compared to the pure PLA. The bending strength reduced to 88–90 MPa from 100 MPa with different weight percentages of wood particles of both species and the lowest values were shown for the samples with 40% of weight percentage. This reduction in bending strength is attributed to the redistribution of binding forces between wood particles and biopolymer. At higher wood filler contents, the bending stiffness of biocomposites significantly increased (50%–90%) with the increasing wood particle content of both species in comparison with the bending stiffness of pure biopolymer. The bending stiffness of pure biopolymer was 3.4 GPa and the stiffness of biocomposites increased to 3.7, 4.7, 5.7, and 6.8 GPa with 10%, 20%, 30%, and 40% of aspen wood weight percentages, respectively, as shown in Figure 4b. Similar, trend was shown by willow wood particles reinforcement, with the highest stiffness of 5.6 GPa with 40% of wood particle content. 

**Figure 5:**
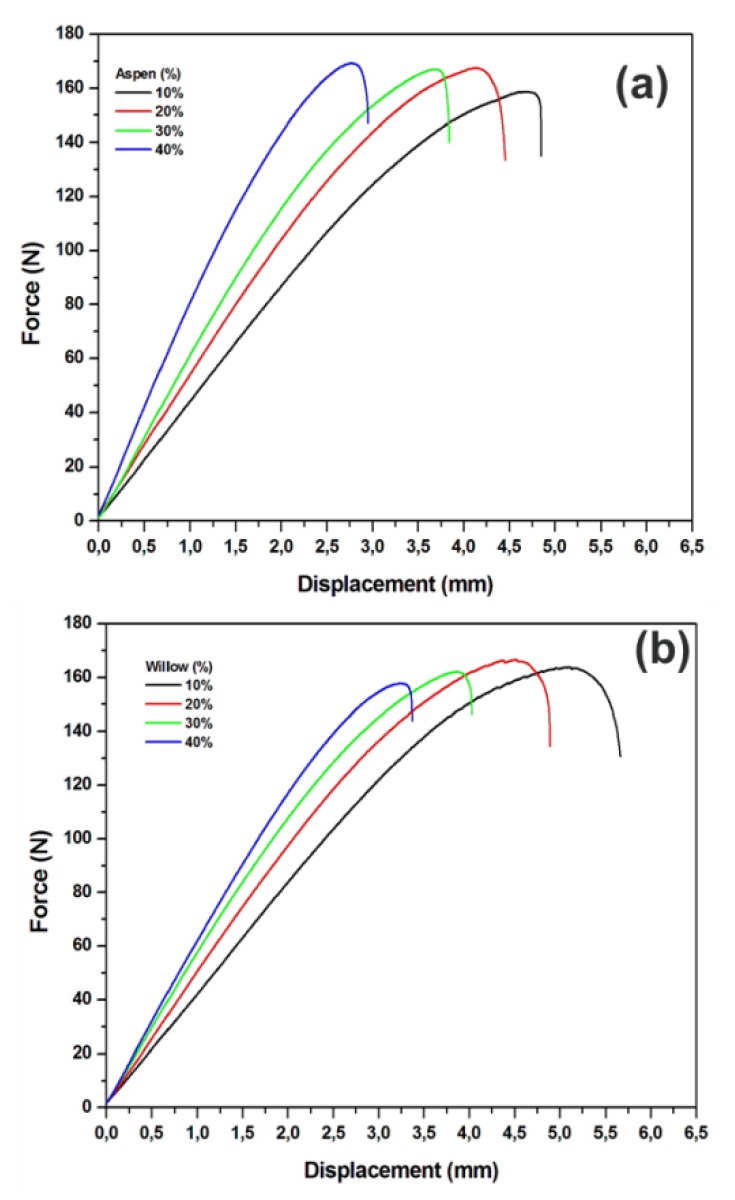
Load-displacement curves of biocomposites for bending strength for (**a**) aspen wood particles and (**b**) willow wood particles.

The stiffness and brittleness of the biocomposites increases with increasing wood particle content (Figure 4b and Figure 5). For all cases, there is a linear increase in the bending stiffness with wood particle content. The load-displacement curves (Figure 5a,b) show that as the content of wood particles increases, the biocomposites show the brittle-behavior with reduced displacement towards bending force.

### 3.4. Brinell Hardness

**Figure 6:**
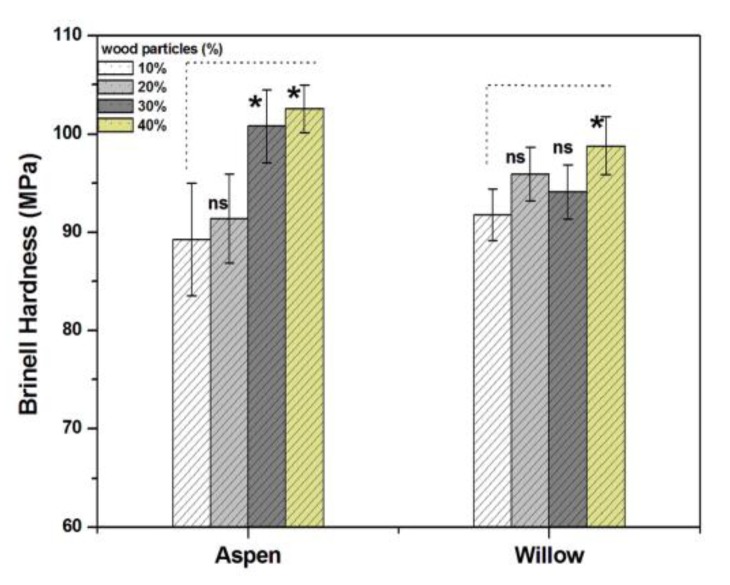
Brinell hardness of biocomposite with different weight percentages of wood particles. (* significant difference at *p* < 0.05 and ns not significant)

The technical hardness is the resistance that a body opposes the penetration of another. The hardness mainly characterizes plastic or mainly elastic deformation, depending on the type of deformation of the materials to be tested [34]. Figure 6 demonstrates the change in the Brinell hardness of biocomposites with wood particles content. The hardness significantly increases with the wood particles content for both wood species. With 10% wood particles, the hardness of biocomposite was 89.27 and 91.80 MPa for aspen and willow, respectively. It was increased to 102.79 and 98.80 MPa for aspen and willow-based biocomposites, respectively, with 40% wood particles content. The average mean Brinell hardness for pure biopolymer was 75 ± 5 MPa, so Brinell hardness of biocomposites significantly increased with increasing wood particles content. Similar results have been reported in literature, where wood particles of other species were used [31,35]. The natural aspen and willow wood have a significantly lower, i.e., 18 ± 3 MPa Brinell hardness when compared to that of biopolymer (75 ± 5 MPa). The hardness of the produced biocomposites increased with increasing wood contents when wood particles were used as fillers in biopolymer.

### 3.5. Correlation between Brinell hardness and Tensile Strength

**Figure 7:**
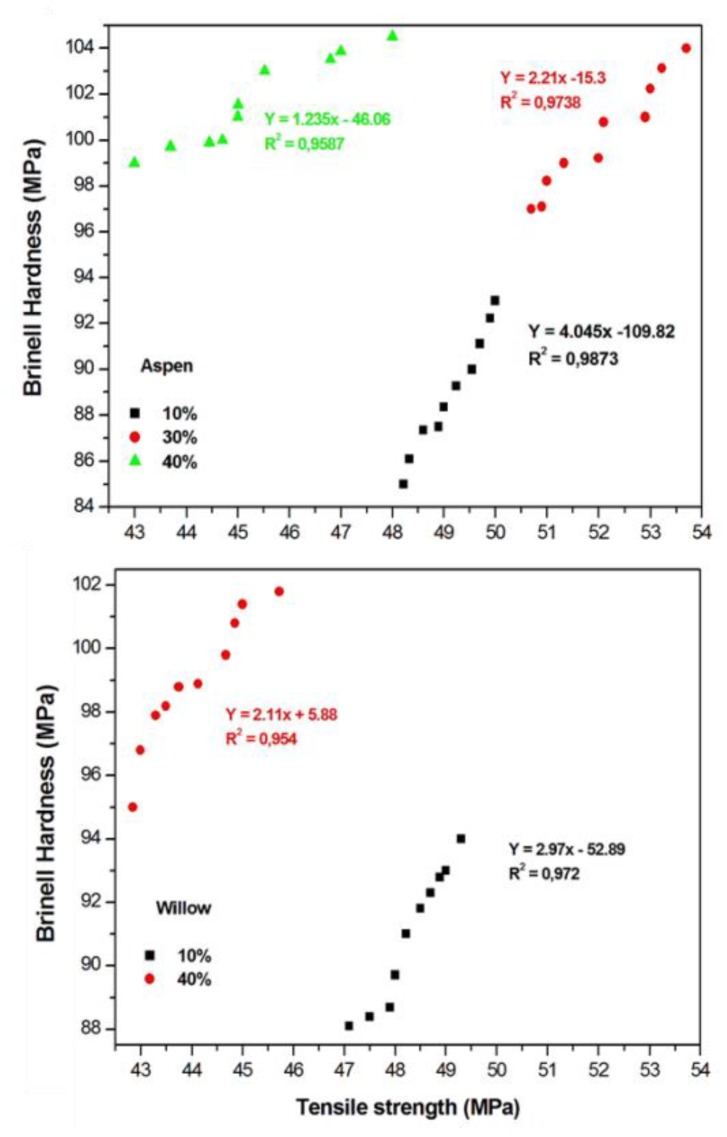
The correlation between Brinell hardness and tensile strength of the biocomposite reinforced with aspen and willow wood particles.

Hardness is of persistent interest to understand the relationships between hardness and other fundamental properties of material [36]. In the present work, the ultimate tensile strength of biocomposite was estimated with different wood particle content of two different wood species. The relationship between the Brinell hardness and the tensile strength of the biocomposite was determined by linear regression analysis and the coefficient of correlation was established between them, as shown in Figure 7. The linear correlation showed a decreasing trend with increasing wood particles content. The highest coefficient correlation R^2^ found was 0.987 for 10% aspen wood particles biocomposite, whereas 10% willow particles filled biocomposite showed R^2^ of 0.972. With 40% wood particles content, the R^2^ was reduced to 0.958 and 0.954 for aspen- and willow-based biocomposites, respectively. A similar value of correlation coefficient was reported [30] for wood plastic composite (polypropylene beech and pine wood mixed wood flour) between the Brinell hardness and tensile strength, where R^2^ significantly reduced when wood flour content of ≥40% was used for polypropylene-based injection molded composite. The interfacial bonding between biopolymer and wood particles might be reduced with increasing wood content, also possibly resulting in lower R^2^. According to literature, this is a typical behavior of thermoplastic composites that are filled with lignocellulosic material [16,18,31,35,37].

### 3.6. FTIR Analysis

**Figure 8:**
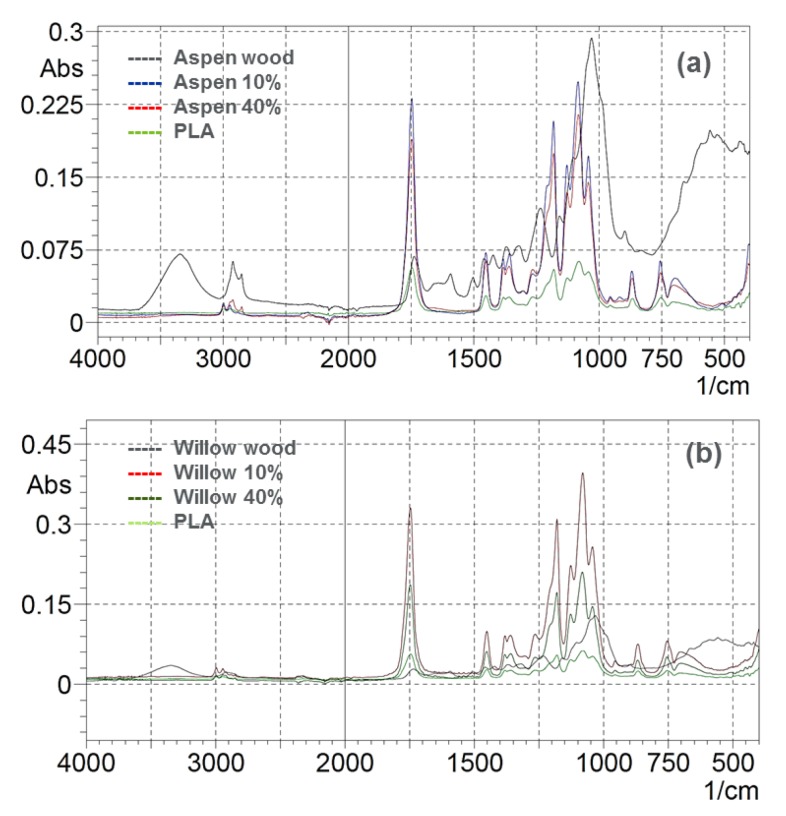
Fourier transform infrared spectrophotometer (FTIR) of wood particles, biopolymer and the biocomposites.

**Figure 9:**
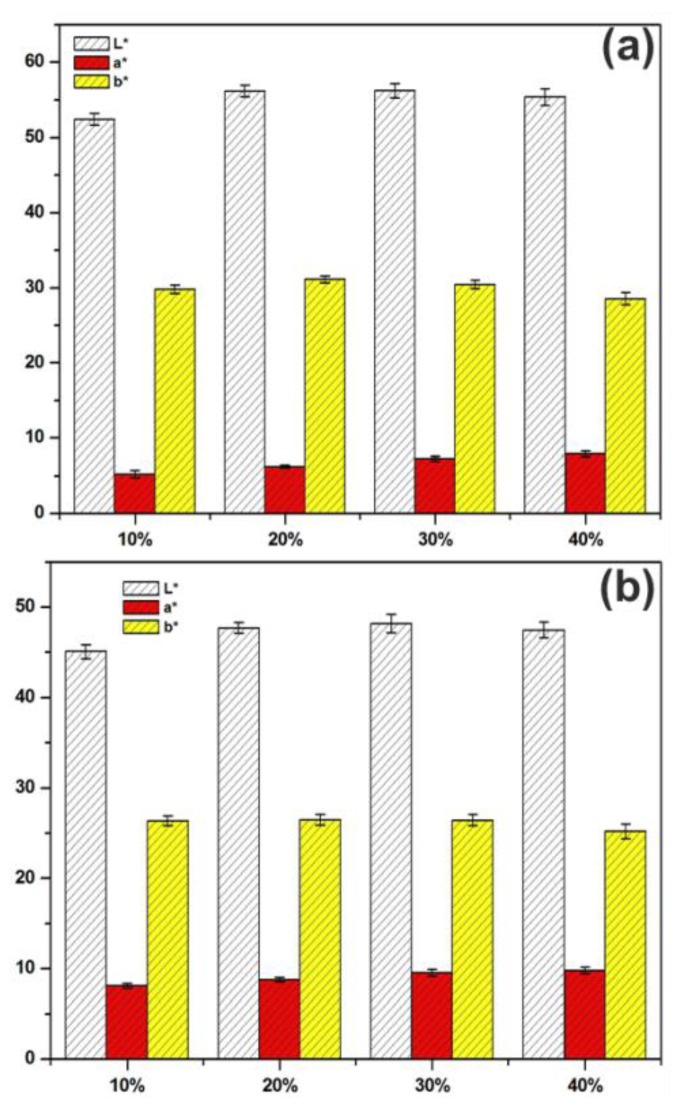
Color measurement of biocomposites with different weight percentage of aspen (**a**) and willow (**b**) wood particles.

Figure 8 shows the typical hardwood FTIR spectra that represent aspen and willow wood with characteristic peaks at 3350 cm^−1^ for O–H stretch (hydrogen-bonded), C–H stretching at 2926 and 2854 cm^−1^; and 1739 cm^−1^ for C=O stretch; 1593 cm^−1^ and 1502 cm^−1^ for aromatic skeletal vibration of lignin; 1234 cm^−1^ for C–O of guaiacyl ring; and, at 1031 cm^−1^ for C–O of primary alcohol and guaiacyl C–H, respectively [38]. The stretching frequencies for C=O and C–O, –CH_3_ asymmetric, –CH_3_ symmetric at 1746 cm^−1^, 2995 cm^−1^, 2946 cm^−1^, and 1080 cm^−1^, respectively, as shown in Figure 9 for biopolymer. The –CH_3_ asymmetric and –CH_3_ symmetric frequencies at 1452 and 1361 cm^−1^, respectively, are the identification of PLA [39]

The biocomposite that was reinforced with aspen and willow wood particles showed the characteristic FTIR peaks (see Figure 9) of biopolymer and aspen wood, as discussed above. The O–H stretch (hydrogen-bonded) at 3350 cm^−1^ of wood was not presented in biocomposite, due to bonding between biopolymer and reactive hydroxyl group of wood. The C–O at 2995 cm^−1^, –CH_3_ asymmetric at 2946 cm^−1^ has shifted to 2926 cm^−1^, 2854 cm^−1^, respectively, when 40% of aspen wood particles reinforced the biopolymer matrix. The peak stretch intensity at 1746 cm^−1^ that represented the C=O stretch of wood and –CH_3_ symmetric of biopolymer significantly increased for biocomposites with increasing wood content.

### 3.7. Color Difference

The lightness values were at the lowest in the groups of 10% of wood particles content, as shown in Figure 9. The low level of the lightness in those biocomposites were probably due to the fact that the glasslike binder of the composite material passed through most of the light and the light was not reflected back to the detector from the sample surface or the surface behind it. The redness of biocomposites increased with increase in wood particles content. Aspen-based biocomposites showed lower redness values as compared to willow-based biocomposites. While yellowness was consistent with different wood particles contents, the difference in yellowness between the wood species was apparent: aspen-based biocomposites showed yellowness values of 30 or more, while willow-based biocomposites had values that were lower than 30.

### 3.8. Microstructure of Biocomposites

**Figure 10:**
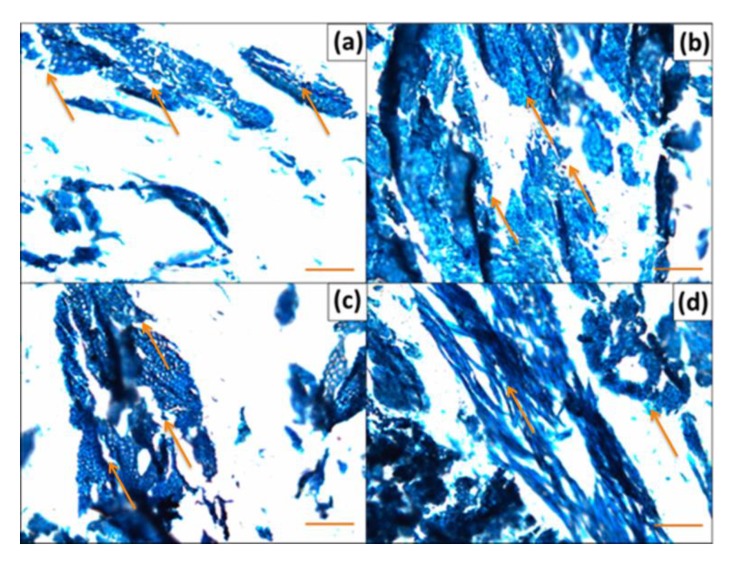
Microstructure of biocomposites with different weight percentage of wood particles mixed to natural binder before the molding process (white color for PLA matrix and blue color for wood particles in PLA matrix): (**a**) 10% of aspen wood, (**b**) 40% of aspen wood, (**c**) 20% of willow wood, and (**d**) 40% of willow wood. Scale bars: 100 µm.

Figure 10 shows the visual appearance of microstructure of biocomposites with different wood particles content of aspen and willow mixed with the PLA matrix. The wood particles were uniformly mixed with the binder and cellular characteristics of wood were well recognizable. The interfacial bonding of wood particles with the biopolymer was homogenous, which resulted in even and high quality of the composite material. It was clear from microstructures that the wood particles were covered by the polymer matrix that could account for good strength performance of biocomposites. Any types of bubbles or voids were not observed between the biopolymer matrix and the wood particles; however, with higher wood particles content, the homogeneity decreased, possibly causing negative impacts on mechanical properties. The arrows in Figure 10 represent the interfacial zone formation between wood particles and PLA materials. At low wood content, the wood particles formed a bigger interface and random distribution with PLA matrix (Figure 10a,c), and wood particles seem to appear ruptured. On the other hand, the high wood content loading into PLA matrix formed smaller interface zone and wood particles appeared to be less ruptured. The higher wood content loading showed lower tensile strength due to the formation of smaller interface zone.

## 4. Conclusions

The present study was focused on exploring the potential utilization of wood raw materials from short-rotation forests, namely aspen and willow, in the production of injection molded biocomposites. The aspen and willow wood particles were mixed as filler in different weight percentages (10%, 20%, 30%, and 40%) into the PLA matrix to produce biocomposites at the industrial scale setup. Biocomposites were analyzed to evaluate their physical (color, density and water absorption, microstructure), chemical (FTIR), and mechanical (tensile strength, bending behavior, and Brinell hardness) properties. The results revealed that the tensile and bending strength initially decreased with 10% weight percentage of wood particles when compared to pure biopolymer, but showed increasing trend with higher wood particles contents. However, the bending stiffness was higher than that of pure PLA already at the lowest wood particles content and increased with the increase in weight percentages of wood particles. The linear correlation between tensile strength and Brinell hardness varies with wood particles content, as the values of the linear coefficient of regression (R^2^) was decreased with increasing wood particles percentage. The microstructure analysis revealed the formation of good interfacial bonding between wood particles and biopolymer, but also variations in the homogeneity with different weight percentages of wood particles. As a conclusion, wood of short-rotation tree species has excellent potential to be used for production of biocomposites and contribute to the sustainable bioeconomy.

## Figures and Tables

**Table 1:** Characteristics of the sample trees in Tuusula, Finland (mean ± standard deviation).

Wood Species	Height (m)	Stem Diameter (cm) *		
		0 m	1.3 m	6 m
Aspen	19.6 ± 0.7	26.1 ± 1.4	20.3 ± 0.6	13.0 ± 0.6
Willow	7.0 ± 2.9	4.7 ± 2.2	1.7 ± 0.5	not measured

* Diameters were measured from two compass directions across the stem and arithmetic mean values calculated.

**Table 2:** Density and water absorption of biocomposites for different weight percentage of wood particles.

Species and Wood Weight Percentage	Density (g/cm^3^)	Water Absorption (%)	
		24 h	4 days
PLA	1.22 ± 0.05	0.20 ± 0.04 ^ns^	0.59 ± 0.07 ^ns^
Aspen 10%	1.23 ± 0.05 ^ns^	0.33 ± 0.04 ^ns^	0.98 ± 0.06 *
Aspen 20%	1.26 ± 0.06 ^ns^	0.53 ± 0.07 ^ns^	1.5 ± 0.15 *
Aspen 30%	1.29 ± 0.05 *	0.75 ± 0.05 ^ns^	2.27 ± 0.2 *
Aspen 40%	1.32 ± 0.04 *	0.90 ± 0.08 ^ns^	2.79 ± 0.18 *
Willow 10%	1.30 ± 0.05 *	0.40 ± 0.04 ^ns^	1.41 ± 0.21 *
Willow 20%	1.31 ± 0.03 *	0.51 ± 0.03 ^ns^	2.01 ± 0.10 *
Willow 30%	1.32 ± 0.04 *	1.25 ± 0.1 *	5.30 ± 0.30 *
Willow 40%	1.33 ± 0.05 *	1.56 ± 0.03 *	6.20 ± 0.27 *

(^ns^—no signifiecant different). (*—Significant difference at *p* < 0.05).
